# Acute post‐infection cerebellar ataxia following SARS‐CoV‐2 infection: A case report

**DOI:** 10.1002/ccr3.5980

**Published:** 2022-06-13

**Authors:** Mehri Salari, Fatemeh Hojjati Pour, Bahareh Zaker Harofteh, Masoud Etemadifar

**Affiliations:** ^1^ Neuro Functional Research Center, School of Medicine Shahid Beheshti University of Medical Sciences Teheran Iran; ^2^ School of Medicine Shahid Beheshti University of Medical Sciences Teheran Iran; ^3^ Department of Neurology, School of Medicine Isfahan University of Medical Sciences Isfahan Iran

**Keywords:** APCA, ataxia, coronavirus, COVID‐19

## Abstract

APCA is characterized as a sudden loss of coordination of muscle movements due to an infection and is the most frequent form of acute cerebellar ataxia (ACA), a common neurological disease in children. We attempt to underline that acute post‐infectious cerebellar ataxia (APCA) can be a post‐COVID complication in children.

## Graphical abstract

cerebellar ataxia can occur after infections. thus it is possible that COVID‐19 contamination cause post‐infectious cerebellar ataxia in children.

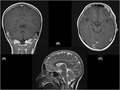

## 1 INTRODUCTION

After two years of spreading SARS‐CoV‐2, a well‐known virus with respiratory symptoms, the economy, society, and public health were greatly affected. COVID‐19 might be life‐threatening, and it causes post‐infection problems such as neurological complications, which have been reported globally.

This virus appears to affect adults more than children, and even neurological complications in adults have been reported more frequently. Although the number of post‐COVID neurological problems is rising due to growing cases of COVID‐19, there is still a shortage of information about post‐COVID‐19 movement disorders in young adults and children.

Studies suggested that nonspecific neurological conditions such as fatigue, headache, myalgia, and weakness are the most common problems in children affected by COVID‐19, and specific neurological complications are uncommon in young adults after recovery from COVID‐19. However, these children are at risk of developing seizures, meningeal signs, and encephalopathy, with variable severity.^1^ There are also some reports of children with encephalitis^2^, ^3^ and acute disseminated encephalomyelitis (ADEM)^4^, ^5^ post–COVID‐19 infection.

Even though ataxia following SARS‐CoV‐2 infection is reported in adults,^6^, ^7^, ^8^ there are just two case reports of APCA after COVID‐19 before age 18.^9^, ^10^ Ataxia is usually a cerebellar disorder with an acute or chronic presentation. As we mentioned before, ACA is generally a childhood disorder and mostly presents after a recent febrile illness.

Herein, we present an immunocompetent young male with acute onset of vertigo and cerebellar ataxia, following coronavirus infection.

## 2 CASE PRESENTATION

A 15‐year‐old boy was referred to our movement disorders clinic with rapidly progressive loss of coordination. He could not walk or stand without help, and he had recurrent fallings to the left side. He reported flu‐like symptoms, including cough, sore throat, runny nose, with a positive reverse‐transcription polymerase chain reaction (RT‐PCR) for coronavirus by nasopharyngeal swab 14 days earlier. After a week, he started to experience positional true vertigo, which continued for 50 seconds and developed gait instability. Family history and past medical history were unremarkable, and there was no pre‐existing cerebellar disorder. There was also no history of COVID‐19 vaccination of any kind.

On examination, he was conscious, alert, and oriented with stable vital signs. All cranial nerves were intact, and there was no speech or sensory dysfunctions. Muscles tone, strength, and deep tendon reflexes were normal and plantar reflexes were down. Examination of the eye movements was normal. Neither meningeal signs nor pathological nystagmus, opsoclonus, or myoclonus were observed.

**FIGURE 1:**
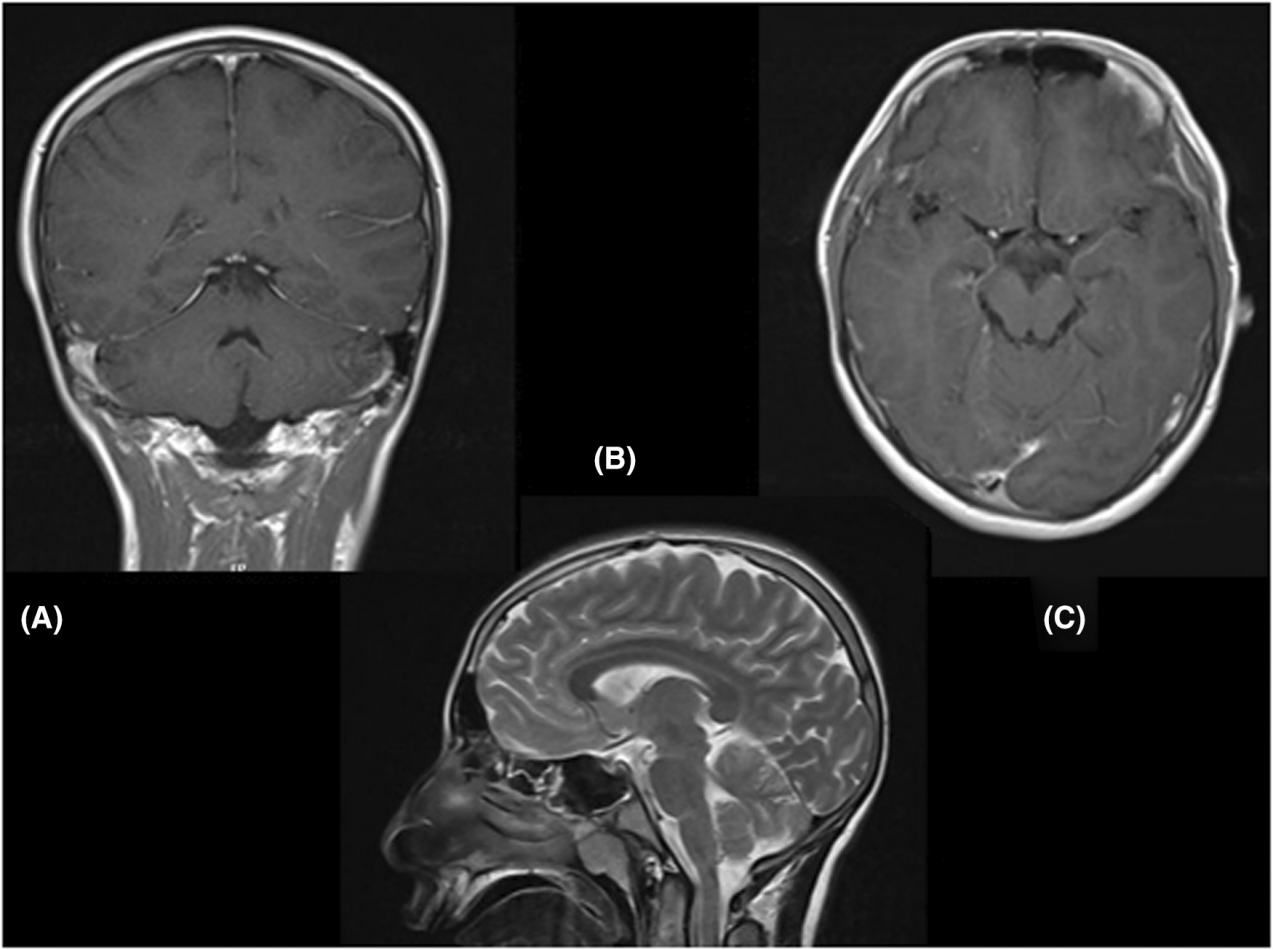
Brain MRI of the patient A: coronal view, B: sagittal T2 view, C: axial T1 view with contrast, showed no abnormalities with or without contrast

Cerebellar examination revealed impaired finger to nose, heel to shin, and rapid alternative movements. Gait was ataxic and he could not walk without help. With ataxic gait and weakened rapid movement (Video S1). The rest of the systemic and neurological examinations were normal. Brain MRI with gadolinium showed no pathological finding. (Figure 1).

Laboratory tests including complete blood count; liver enzymes, renal functioning, thyroid function tests; antistreptolysin titer; inflammatory markers including Erythrocyte sedimentation rate and C reactive protein; 24 h urinary copper and serum ceruloplasmin; complete autoimmune screening: immunoglobulins subpopulations, complement C3 and C4 fragments, antinuclear antibodies (ANA), extractable nuclear antigens (ENA), antineutrophils cytoplasm antibodies (ANCA), antibeta 2 microglobulin antibodies, antithyreoperoxidase antibodies, antiendomysium antibodies, anticardiolipin antibodies, antiphospolipids antibodies (PLP), lupus anticoagulant; autoimmune panel in CSF and serum, and CSF OCB, were negative.

A lumbar puncture was performed, and CSF testing indicated a negative culture and normal analysis. Testicular sonography, abdominal CT, and chest CT were unremarkable.

The diagnosis of post infection ataxia was made based on the clinical presentation, laboratory findings, and excluding all other causes of ataxia.

He was treated with intravenous methylprednisolone (1 g/day) for 5 days followed by oral prednisolone (50 mg/day), and symptoms begin to improve rapidly.

## 3 DISCUSSION

The evidence suggests that it is needed to keep close attention on people after acute SARS‐CoV‐2 infection, because some clinical manifestation may occur even after the recovery. Therefore, it is essential to recognize which problems will arise after the infection. Moreover, the effect of the pandemic on a vulnerable group of people like children is necessary for the governments' adaptation since early treatment allows rapid recovery. We report a case with acute cerebellar ataxia as a possible treatable complication in pediatric patients after COVID‐19 infection.

However, there has been more evidence for ataxia in adults post–COVID‐19,^7^, ^8^as far as we know, there are just two case reports of APCA caused by SARS‐CoV‐2 infection. One in a 13‐year‐old boy who developed cerebellar ataxia 10 days after classic manifestations of COVID‐19 and a positive PCR test for coronavirus. He was treated with intravenous methylprednisolone for 5 days and improved over 20 days.^10^

The other case is a 5‐year‐old toddler who suffered from diabetes mellitus type 1. After 8 days from mild manifestations of coronavirus, he showed impaired balance, shaking hands, and double vision. And after 3 days, he presented with ataxic gait and mild dysarthria. Also, his cerebellar examination was impaired. His symptoms resolved after 2 months without any treatment.^9^

Although the number of these cases is rising, there is currently no definition for this condition and the exact mechanism by which SARS‐CoV‐2 may cause cerebellar dysfunction remains unclear until now. But evidence suggests, according to the onset of neurological manifestations after recovery from COVID‐19 contamination and the perfect response of these symptoms to immunotherapy, this phenomenon can be immune‐mediated.^6^ Thus, coronavirus probably activates excessive immune system with cytokine storm, or cause immune response cross‐reactive to cerebellar autoantigen to hurt cerebellum.^8^ Consequently, these neurological disorders occur even after recovery from COVID‐19 infection, and they can be treated with immunological medications such as steroids. It must be noted; despite near‐complete remission after a few months, clinicians should be aware of this possible manifestation in children recently affected by COVID‐19 to start treatment as soon as possible for the best result.

In conclusion, this case report introduces a rare neurological complication in children that suggests a link between COVID‐19 and acute post‐infection cerebellar ataxia. Further research is needed to prove this association and show which pediatric patients are at risk for developing ataxia.

## AUTHOR CONTRIBUTIONS

Mehri Salari involved in conception, organization, and execution of the research project. She also involved in writing the first draft of the manuscript. Fatemeh Hojjati pour wrote the first draft of the manuscript. Bahareh Zaker Harofteh executed the research project. Masoud Etemadifar involved in conception, organization, execution of the research project. He also reviewed and involved in the critique of the manuscript.

## CONFLICT OF INTEREST

The authors have no financial conflicts of interest.

## ETHICAL APPROVAL

Written consent was obtained from patient's parents for using his video for publication.

## CONSENT

Written informed consent was obtained from the patient's parent to publish this report in accordance with the journal's patient consent policy.

## Supporting information


Video S1

## Data Availability

The data that supports the findings of this study are available in the supplementary material of this article.
